# Understanding Apple Attribute Preferences of US Consumers

**DOI:** 10.3390/foods11020166

**Published:** 2022-01-09

**Authors:** Rombach Meike, David L. Dean, Tim Baird

**Affiliations:** 1 Department of Land Management and Systems, Lincoln University, Lincoln 7647, New Zealand; 2 Department of Agribusiness and Markets, Lincoln University, Lincoln 7647, New Zealand; david.dean@lincoln.ac.nz (D.L.D.); tim.baird@lincoln.ac.nz (T.B.)

**Keywords:** apple varieties, attitudes, knowledge, US consumers

## Abstract

Apple preferences of US consumers are widely explored. However, the key factors that drive the importance that US consumers place on apple attributes are rather unexplored. To fill this literature gap, an online survey with 383 US apple buyers was conducted. A two-step analysis consisting of descriptive statistics and partial least squares structural equation modelling indicates that subjective knowledge was the most important factor, determining both the discernment of buyers and attitudes towards US fruit growers. Objective knowledge and sociodemographic factors, other than education, were not found to have any impact. The discernment of a buyer and their ability to distinguish apple varieties had the greatest impact on the importance that US consumers placed on physical and commercial product attributes. It was also found that attitudes towards growers impacted on the importance which consumers place on both types of attributes. Given that consumer attitudes were shown to be a strong driver of their buying preferences, growers and grower associations should also consider highlighting the positive health and societal benefits that their products provide.

## 1. Introduction

Fresh apples are a commonly consumed and widely available product in food markets around the world [1,2]. They are valued for their health benefits, which include being rich in Vitamin C and phenolic compounds [3,4]. In today’s food markets, consumers have the choice to either buy domestically produced apple varieties or those that are sourced from overseas [5]. For the US, New Zealand is one of these overseas sources. New Zealand’s reputation as a global leader in apple breeding, production and export makes sourcing apples from there attractive for overseas markets [6,7,8]. Seasonality can mean that apples grown in the US are insufficiently available, and this lack of supply is filled with Southern-Hemisphere produce. The main share of US apples is produced in Washington, Michigan, New York, Oregon and Pennsylvania [9]. These states are known for their large-scale production, technological innovation and being the home to many traditional and robot-ready apple orchards [10,11]. Traditional varieties, such as Honeycrisp, Fuji, Granny Smith, Red Delicious, and Golden Delicious, as well as club varieties, such as Jazz, Pink Lady, Sweet Tango, and Ambrosia [12,13], are popular among US consumers. This development towards modern apple varieties, as well as an increase in the level of investment in orchards, is evidence that the US apple industry is consumer-oriented and striving to provide products that satisfy the needs and wants of fruit consumers [1,14].

Apples as a horticultural consumer good are comprised of various product attributes, some of which may have varying levels of importance for consumers. Relevant consumer attributes possessed by fresh apples include the colour of the skin, shape, aroma, apple variety, texture and the length of their shelf life [15,16,17,18]. This latter attribute is particularly important, as even though apples have good storing qualities, they are ultimately perishable [19,20,21]. Colour and appearance are crucial in retail situations as they attract the consumer’s attention. Colour often serves as a cue for fruit quality; consumers commonly attempt to estimate the texture of apples as this gives them an indication of the taste [22]. Extant literature in this area classifies consumers into two main categories: those who prefer firmness, juiciness, and bit of acidity in apples, and those that who like sweeter, but less firm apples [23]. In addition to these product attributes which are inherent to the apple (intrinsic attributes), consumers are also interested in commercial attributes, such as price, packaging, branding, country of origin, and sustainability [24,25,26]. These are linked to the production, distribution, and presentation of apples (extrinsic attributes) [27,28,29,30,31]. Although early studies on horticultural and agricultural products have emphasised the importance of intrinsic attributes for consumers, more recent studies show that for agricultural and horticultural products external attributes are equally important for consumers [2,32,33,34,35]. Consumer choices regarding apple attributes, as well as the willingness to pay for fresh or processed apple products has been intensively studied in the US [12,36,37]; Consumer choice relies on a trade off between bundles of intrinsic and extrinsic product attribute; these include aspects of consumers personal backgrounds, including their sensory preferences and attitudes [38]. However, key-factors which lead to the determination of apple preferences are not as widely studied. In the following sub sections these factors are explained in more detail as they underpin the conceptual framework for this study. US consumers’ objective and subjective knowledge, as well as their sociodemographic backgrounds, their discernment as a buyer and their attitudes towards apple growers are likely to be key factors in determining the importance that US consumers place on physical and commercial apple attributes.

### 1.1. Objective and Subjective Consumer Knowledge

Horticultural products, such as apples are information-intensive products [38], as many varieties exist. The type of production, such as organic and conventional production, influence orchard management, and ultimately the final product [39,40]. In order to receive information about food quality, food safety, and whether a product is local or not, consumers use different intrinsic and extrinsic attributes [36,40,41,42]. However, identifying this desired information is often not a straightforward task for consumers [35].

Understanding consumers’ needs and wants, their behaviors and knowledge is essential for marketers in the US food retail industry to successfully target different fruit consumer segments. In terms of consumer knowledge, different types of knowledge, such as subjective and objective knowledge need to be distinguished [43].

Subjective knowledge is known as self-reported knowledge, and relies on consumers’ self-assessments and their perceptions, which may be incorrect [44]. Objective knowledge is correct and accurate knowledge, which is assessed through testing, and is stored in long-term memory of the consumer [44]. Objective knowledge can be obtained intentionally or unintentionally. Intentional knowledge occurs when consumers make a conscious effort to learn specific product information, while unintentional knowledge occurs when consumers are exposed to stimuli [44].

Subjective and objective knowledge are equally important when assessing consumers’ apple knowledge [45]. However, the literature on both types of knowledge is inconclusive. Some studies emphasise the correlation between subjective and objective knowledge, and suggest that both types of knowledge are interconnected; other studies have shown that they can be different, and stress that subjective and objective knowledge are only inconsistently correlated [46]. Nevertheless, there is agreement that consumer knowledge impacts their preferences, and ultimately their buying behaviour [45,46].

### 1.2. Socio-Demographics

Various studies discuss the socio-demographic backgrounds of US consumers who purchase fresh apples; however, there is no consensus in the body of literature on these socio-demographic backgrounds, and even less for preferences for specific apple attributes. Some studies indicate that buying apples is associated with gender, age, high income, and education [47]. Being female is also an important factor, as it has been reported that the majority of grocery shoppers in the US are women [12]. Other studies diverge from socio-demographic information as key factors. Instead, they highlight dietary preferences, attitudes, and lifestyle choices [48,49]. Given that there are differences in apples being offered in food retail across regions in the US, apple variety preferences across states or regions are very heterogeneous [12,50,51].

Studies being specifically dedicated to intrinsic and extrinsic apple attributes highlight that consumer preferences are heterogenic, and that consumer backgrounds are equally diverse [12,52]. Preferences for physical attributes, such as appearance, texture and taste are difficult to associate with socio-demographic backgrounds, This is due to consumers who are at the point of sale or who are participating in sensory experiments not always being able to correctly distinguish amongst these attributes, which makes it difficult to for the consumer to express their preferences [52,53,54]. In addition, various studies focus only on single attributes, or a few selected attributes [52,53,54].

### 1.3. Apple Buyer Discernment

For the US food retail industry, as well as for the horticultural industry, it is important to know consumer preferences for new and existing varieties, as well as their ability to distinguish varieties [52]. This allows businesses to offer products that consumers need and want, and enables marketers to differentiate their products from existing ones. Very few studies have focused on the perception of apple varieties and the ability of consumers to distinguish them [52]. Studies which have shown that consumers are necessarily able to distinguish apple varieties have found that mostly neophobia or neophilia determines preference or aversion towards new apple varieties [12,52]. In the US, new varieties are often termed as club varieties [12]. Club varieties are subject to patent-protection. Growers who are part of the club have exclusive rights to produce and market the club variety as stipulated by a licensing contract. This includes both fruit quality and quantity [55]. Common examples of club varieties on the US market are ‘Jazz™’, ‘SnowSweet^®^’, ‘Sweet Sixteen’, ‘SweeTango^®^’, ‘Zestar!™’, and ‘Pink Lady^®^’ [12]. Examples of more traditional varieties are ‘Red’ and ‘Golden Delicious’, ‘Granny Smith’, ‘Fuji’, ‘Honeycrisp’, ‘McIntosh’, ‘Cripps Pink’ [13]. Given that the majority of consumers do not possess a good varietal knowledge, marketing promotions, such as tasting experiences which offer free samples coupled with promotional materials regarding varietals are crucial to improve the ability of consumers to distinguish amongst different varieties [55].

### 1.4. Attitudes towards Growers

Attitudes refer to a learned tendency to evaluate things, people, or events either favourably or unfavourably [46]. Attitudes towards horticultural and agricultural production and growers are quite diverse. Although some studies report consumer trust and positive attitudes towards products, growers, and production processes, others report concerns, distrust, perceived risks, and negative attitudes [46]. Issues discussed in this context and which influence attitudes towards growers are challenges regarding production, technological innovation, disease and pest management, payments, treatment of labour and labour conditions, environmental impacts, and resource usage [56].

### 1.5. Objective and Hypotheses

**Figure 1:**
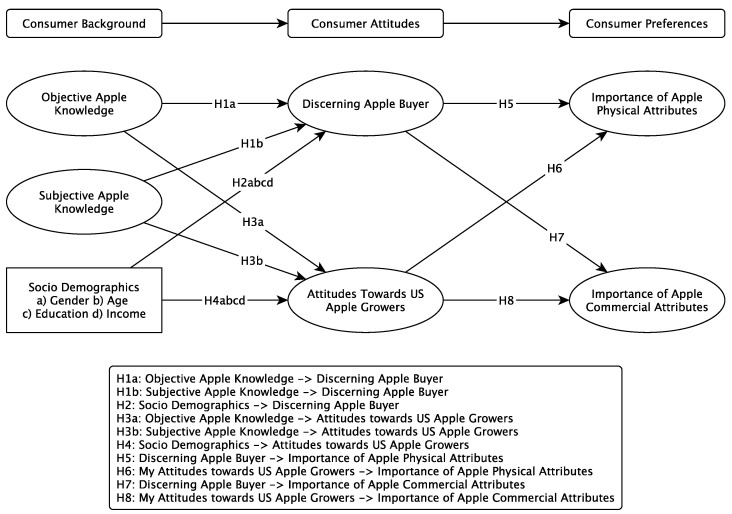
Conceptual Framework.

Using the extant literature in this topic area as a foundation, this study aims to explore the drivers of US consumers apple attribute preferences. The theoretical framework (see Figure 1) is based on the literature presented. It is suggested that the importance that US consumers place on apple attributes is likely to be influenced by their socio-demographic background, their objective and subjective knowledge, their discernment as a buyer, and their attitudes towards horticultural growers. Consumer specific information, such as socio-demographic background and knowledge, can influence both the consumers’ ability to distinguish apple varieties and their respective attitudes towards horticultural products, such as apples, and the growers of these products. Given that an attitude refers to a tendency to that is expressed by evaluating an entity either positively or negatively, consumer attitudes can impact the importance they place on apple attributes. Depending on their knowledge and attitudes, consumers may evaluate intrinsic and extrinsic product attributes either favourably or unfavourably [12,45,46,52,55,56].

**Hypothesis** **1** **(H1).** *Being a discerning apple buyer is likely to be positively impacted by (a) objective knowledge, and (b) subjective knowledge.*

**Hypothesis** **2** **(H2).** *Being a discerning apple buyer is likely to be positively impacted by (a) gender, (b) age, (c) education, and (d) income.*

**Hypothesis** **3** **(H3).** *Attitudes towards US apple growers are likely to be positively impacted by (a) objective knowledge, and (b) subjective knowledge.*

**Hypothesis** **4** **(H4).** *Attitudes towards US apple growers are likely to be positively impacted by (a) gender, (b) age, (c) education, and (d) income.*

**Hypothesis** **5** **(H5).** *The importance that consumers place on physical apple attributes is likely to be positively impacted by their discernment as an apple buyer.*

**Hypothesis** **6** **(H6).** *The importance that consumers place on physical apple attributes is likely to be positively impacted by their attitudes towards US growers.*

**Hypothesis** **7** **(H7).** *The importance that consumers place on commercial apple attributes is likely to be positively impacted by their discernment as an apple buyer.*

**Hypothesis** **8** **(H8).** *The importance that consumers place on commercial apple attributes is likely to be positively impacted by their attitudes towards US growers.*

## 2. Material and Methods

### 2.1. Research Design and Data Collection

In October 2021, an online survey was conducted to receive information about US consumers’ apple preferences. The online survey software Qualtrics and Amazon Mechanical Turk, a crowdsourcing marketplace were used to distribute the survey [57,58]. The survey was designed to obtain information such as respondents’ socio-demographic backgrounds, as well as respondents’ knowledge about apples and apple production in the US. It was also designed to examine their perceptions and attitudes towards traditional and club varieties, as well as towards US growers. To participate in the survey, respondents needed to be 21 years old and reside in the US. The data collection resulted in 461 responses, of which 400 were apple consumers. Among these 400 consumers, 383 delivered complete responses that were suitable for analysis. The minimum sample consisted of 196 male and 187 female respondents targeted to be apple buyers. The required minimum sample size of 385 people was estimated via power analysis. The sample of 383 US apple buyers who completed the survey is appropriate for an analysis using descriptive statistics and partial least squares structural equation modelling (PLS-SEM), as the latter approach is particular suitable for small samples [59,60,61]. Applying the “10-times rule” is common convention in PLS-SEM. The rule states that the sample size needs to be greater than 10 times the maximum number of inner or outer model links pointing at any latent variable in the model [59,60,61].

Although many of the constructs have already been discussed in the literature, previous studies offer very few validated scales to adopt for the current research. Therefore, items were created from the relevant concepts proposed in the existing body of literature within this topic domain.

The importance of apple attributes (8 items) was measured using a 7-point importance scale (1 = Extremely unimportant to 7 = Extremely important), and were divided into physical attributes (4 items) and commercial attributes (4 items). The category of discerning apple buyer (7 items) was measured using a similarity scale (1 = very dissimilar to 7 = very similar) assessing perceived similarity between a number of apple varieties. Subjective apple knowledge (4 items) and attitudes towards US apple growers (6 items) was measured using 7-point Likert scales (1 = strongly disagree to 7 = strongly agree). The subjective apple knowledge scale included statements about apple understanding, confidence, and knowledge relative to others. The attitudes towards US apple growers included statements about their traditions, contributions, and social pressures. Finally, objective apple knowledge consisted of a series of multiple-choice questions about domestic and foreign apple production and labelling. The items consisted of 5 factual questions about these issues, and responses were scored with 1 for correct and −1 for incorrect resulting in an index with a possible range of −5 to +5.

### 2.2. Data Analysis

SPSS was used in the management of the data and the calculation of descriptive statistics. PLS-SEM analyses, using SmartPLS, were employed to identify the significant determinant factors of the importance that US consumers place on physical and commercial apple attributes. PLS-SEM methods are widely applied in the social sciences [59], and they generally combine three analytical approaches; regression analysis, path analysis, and principal component analysis [60,61]. PLS-SEM methods are particularly appropriate in situations where researchers are dealing with small sample sizes, non-normally distributed data, and when explorative models contain causal dependencies amongst latent constructs [60]. A two-step approach was applied throughout the PLS-SEM analysis, starting with what is called model measurement (analysis of the inner model) followed by model structure (analysis of the inner model) [60,62].

Following the research of Hair et al. (2019), model measurement aims to verify that model is measuring the constructs correctly both within and between measurement scales. In order to enable this, reliability and validity testing was utilized [60] where loadings greater than 0.4 confirm that items contribute to their appropriate scale. Average variance extracted (AVE) scores greater than 0.5 indicate that the scales have met the variance of their proposed items. Checks for the reliability or internal consistency of scale items were carried out through utilizing both Cronbach’s Alpha (>0.6) and composite reliability (>0.6) measurements [63].

An evaluation of cross-loadings, as well as using the Fornell–Larcker criterion is a common approach to determine discriminant validity; this, in turn, confirms that scales are measuring distinct concepts and items belong to those scales [60,63]. Cross-loading checks ensure that a higher correlation is able to be obtained for all items with their appropriate factor, and this, subsequently, can make sure that other factors are not interfering with these results. The Fornell–Larcker criterion is satisfied when item correlations with the square root of the individual constructs’ AVE is greater than correlations with other constructs [61,64]. Following the work of Henseler et al. (2015), discriminant validity is also measured using the Heterotrait–Monotrait ratio of correlations criterion (HTMT), and this is confirmed when a threshold value of 0.9 is achieved [65]. Finally, the variance inflation factor (VIF) can also be employed to search for high levels of multi-collinearity, and when this is under 5, then the data are suitable for further analysis [60].

Step 2 in the PLS-SEM analysis, model structure, examines the predictive relevance of the model, the accuracy of its structural fit, alongside its explanatory power [61]. Although Hair et al. (2017) contend that SEM-PLS do not lend themselves to model fit indices [63], normal practice is to report both Normed Fit Index (NFI) and Goodness of Fit (GoF), where both NFI and GoF scores vary on a scale from 0 to 1. Larger scores are indicative of a better fit. Smaller Standardised Root Mean Square Residual (SRMR) also indicates a better fit; however, values of more than 0.10 are noted as being problematic, while values which are of 0.08 or less are viewed as being acceptable.

The individual and average variance explained (R^2^) of the dependent variables provides a foundation for the explanatory power of the model. Following Hair et al. (2017), values need to be interpreted as follows: while 0.75 classifies as sizeable, a variance of 0.5 is considered to be moderate, while a variance of 0.25 is considered to be weak [61]. The Stone Geisser criterion (Q^2^) estimates the predictive validity, which should be larger than zero [61]. Indications of medium and large predictive accuracy are shown by Q^2^-values that are larger than 0.25 and 0.5 [61].

## 3. Results

The descriptive statistics of the sample are displayed in Table 1. The median respondent was aged between 25 and 34 years, had obtained a bachelor degree, and earned an annual pre-tax income ranging between USD 25,000 to USD 50,000 per year. Additionally, the other scale measured in the model was the objective apple knowledge score, which had a mean of 1.02, a range of between −4 to +5, and a standard deviation of 1.834.

The measurement model assessment included the use of reliability to test the model constructs, as well as the use convergent and discriminant validity to conduct further checks. All items achieved a factor loading of well above the minimum of 0.4, indicating their suitable contribution to the scale (see Table 2). Reliability was confirmed by both the Cronbach Alpha and composite reliability scores being above 0.6. Convergent validity was also indicated by AVE scores being higher than 0.5 for all the scales. Given that all indicators were within acceptable ranges, the requirements of construct reliability and validity were considered satisfactory [61].

Both the Fornell–Larker criterion and Heterotrait–Monotrait (HTMT) ratios were utilized to test discriminant validity, with the requirements for discriminant validity being met for all of the variable constructs (see Table 3). The square root of each constructs’ AVE was found to be higher than its correlation with other constructs. HTMT ratios are all less than 0.90, with the exception of the HTMT ratio between the importance placed on physical apple attributes and the importance placed on commercial apple attributes (1), which is a higher ratio than that which is recommended. However, this does not represent a problem because the two constructs both measure the apple attribute importance, with one construct being intrinsic and the other extrinsic to the product. Additionally, the largest VIF was 1.338 and the average VIF was 1.158, indicating that there were no problems with multicollinearity [58].

**Figure 2:**
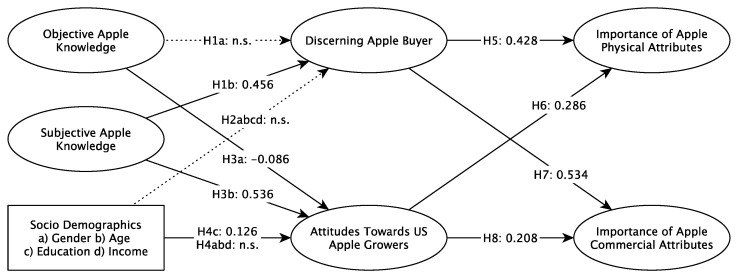
Results of the conceptual model.

The conceptual framework and its overall structure was tested, resulting in a Goodness of Fit of 0.43 and a Normed Fit Index of 0.676. A Standardised Root Mean Square Residual of 0.074 was also achieved, and this indicated that adequacy of the overall model fit. The explanatory and predictive power of the conceptual model was also tested, and this resulted in average R^2^/Q^2^ values of 0.349/0.293, which indicates that the model has overall weak/moderate explanatory power and moderate predictive relevance. However, some parts of the model were found to be stronger than other parts. The R^2^/Q^2^ scores of 0.248/0.336 for discerning apple buyers would be considered weak in their explanatory power and moderate in their predictive relevance, but the score of 0.440/0.216 for importance placed on commercial apple attributes, and 0.388/0.247 for importance placed on physical apple attributes indicate weak/moderate levels of explanatory power and small predictive relevance. The score of 0.321/0.372 for attitudes towards US growers would be considered to have moderate explanatory power and medium predictive relevance. The structure of the model was confirmed to be fit for hypothesis testing due to the adequate model fit, the weak to moderate explanatory power, and the weak to medium predictive accuracy. Table 4 and Figure 2 show the results of the hypothesis testing.

## 4. Discussion

The present study explored key factors determining the importance that US apple consumers placed on physical and commercial apple attributes. Overall, the proposed model was found to have an adequate fit and good explanatory and predictive power. The results emphasised the importance of subjective knowledge as the most important factor determining the discernment of buyers and attitudes towards US growers. Objective knowledge and sociodemographic factors other than education were not found to have any impact. The discernment as a buyer and the ability to distinguish apple varieties had the greatest impact on the importance that US consumers placed on physical and commercial product attributes. Additionally, attitudes towards growers impacted the importance consumers placed on both types of attributes.

Subjective knowledge is a strong driver of buyer discernment and can be explained by the fact that consumers who feel that they have good or expert apple knowledge may see variations across apple characteristics. These findings echo recent studies of wine consumer knowledge presenting different types of consumers with varying levels of knowledge [38,44].

The results concerning buyer discernment as a predictor of the importance that US consumers place on physical and commercial attributes can be explained as follows; being able to distinguish varieties implies a certain degree of apple variety knowledge in terms of appearance, taste, texture, and smell. The distinction between more traditional varieties and club varieties is made through commercial features of the product [55]. This is commonly achieved through branding, and signalled to the consumer through slogans, pricing, labelling, and packaging.

In a similar manner, attitudes towards US growers determine both the importance placed on physical and commercial apple attributes. This supports the findings of Robertson et al. (2018) which showed that consumers with self-reported expertise focused on news sources and other information regarding apple production, and were likely to form stronger attitudes around production processes [66]. In a consumers’ mind growers may represent and oversee products, the types of production and production processes, and their respective impacts on people and environment. The types of production and the production process used impact both the physical product and the commercial factors of the product alike. For this reason, consumer attitudes towards growers may be seen as strong driver.

### 4.1. Practical Implications

Results of this research are relevant to different actors within the horticultural industry and the US food retail industry. US fruit growers may wish to capitalise on the findings related to buyer discernment. Growers can contribute to consumer education and help to improve the varietal knowledge which consumers possess; this may, in turn, lead to brand loyalty. In local shops, at the farm gate, or in online stores information about taste, consumption and processing is very important. The identification of apple variants, and the highlighting of an apples’ pedigree may be interesting for consumers. This could also help to improve the varietal knowledge that consumers have, and help them make more informed decisions when buying apples. Retailers may contribute in a similar manner, highlighting varietal information, the type of production and production processes used via quick response codes.

Given that consumer attitudes are a strong driver of their preferences, growers and grower associations should consider action outlining positive health and societal contributions which exist beyond food production. This impacts the public perception of the horticultural industry positively, and allows growers to both establish and keep their social licence to operate as fruit growers.

### 4.2. Limitation and Suggestions for Future Research

The data used in this study originate from the marketplace Amazon-Mechanical Turk (Mturk) [56]. Within this marketplace, wages of survey respondents are driven by market forces through the requesters posting their surveys and offering compensation. Ten years ago, Mturk was criticised for its low pricing, labour issues, and data quality; however, in 2021, the crowdsourcing platform is now widely used in the social sciences for data collection. Mturk samples and have been found to be equal to other forms of convenience samples [56,57,58]. A sample of Mturk workers is unlikely to be of the same quality than a representative national sample, but comparable to college samples and other forms of convenience samples [56].

Future research could focus on consumer preferences for apple trees and canopy shapes, as self-sufficiency and do-it-yourself are currently popular horticultural trends within gardening as a result of the COVID-19 pandemic, and which are predicted to remain popular [67,68].

In addition, studies may choose to address apple attribute preferences, such as those explored by Bir et al. (2021) [69] who studied attributes of horticultural products using a best-worst approach, evaluating the trade off consumers make among products consisting of various attribute bundles. This study focused on a pictorial experimental design [69]. A similar approach may also be suitable when studying apples and consumers who are novices, or when conducting research with children. Combining the best–worst methodology and latent class analysis on apple attributes would be appropriate in such a study context. The combination enables us to explore the trade offs consumers make when choosing products, and building consumer classes according to common preferences. Within this context the research may consider varieties that are utilized for processing, such as varieties used in juicing and baking, as well as eating fresh. Additionally, a cross-country comparison investigating extrinsic and intrinsic attributes may be promising.

Further research could be framed within the context of buying local and focus on consumers’ willingness to pay for apples produced in specific states or regions in different online scenarios. Such a study could be positioned within the context of connected consumerism, a trend gaining increasing importance due to the COVID-19 pandemic [67]. Connected consumers buy and communicate online with local businesses, are interested in production practices, and are also concerned about the impact of the coronavirus pandemic on both communities and businesses [67]. Examination of the level of connectedness between consumers and apple growers is an area which currently remains unexplored.

## 5. Conclusions

This study focused on attribute preferences of US fruit consumers, and, more specifically, the factors that determine these preferences. Results show that subjective knowledge was the most important factor determining the discernment of buyers and attitudes towards US growers. Objective knowledge was not found to have any impact, while only education as a sociodemographic factor had impact. The discernment as a buyer and the ability to distinguish apple varieties had the greatest impact on the importance that US consumers placed on apple attributes. Additionally, attitudes towards growers impacted the importance consumers placed on intrinsic and extrinsic apple attributes.

## Figures and Tables

**Table 1:** Sample description.

	Freq	%	Median	StDev
Age				
Under 21	2	0.5		
21–24	16	4.2		
25–34	215	56.1	✓	0.940
35–44	104	27.2		
45–54	27	7.0		
55–64	14	3.7		
65+	5	1.3		
Total	383	100		
Education				
Did not finish high school	6	1.6		
Finished high school	46	12.0		
Attended University	40	10.4		
Bachelors Degree	223	58.2	✓	0.927
Postgraduate Degree	68	17.8		
Total	383	100		
Household Annual Income				
USD 0 to 24,999	80	20.9		
USD 25,000 to 49,999	117	30.5	✓	1.141
USD 50,000 to 74,999	119	31.1		
USD 75,000 to 99,999	40	10.4		
USD 100,000 or higher	27	7.0		
Total	383	100		
Gender				
Male	196	51.2	✓	0.501
Female	187	48.8		
Total	383	100		
US Geographical Distribution				
North-East	83	21.7		
Mid-West	133	34.8		
South	90	23.5		
West	77	20.1		
Total	383	100		

**Table 2:** Scale loadings, reliabilities, and convergent validity.

Scales and Items	Factor Loadings	Cronbach’s Alpha	Composite Reliability	Average Variance Extracted
Discerning Apple Buyer		0.836	0.877	0.504
How similar are Pink Lady and Cosmic Crisp	0.741			
How similar are Granny Smith and Royal Gala	0.731			
How similar are Pink Lady and Cripps Pink	0.706			
How similar are McIntosh and Braeburn	0.749			
How similar are Zestar! and Sweet Tango	0.718			
How similar are Fuji and Red Delicious	0.639			
How similar are Red Delicious and Golden Delicious	0.680			
Importance of Apple Commercial Attributes		0.701	0.817	0.527
Importance of—Price	0.702			
Importance of—Labelled as sustainable	0.719			
Importance of—Labelled as traditional varieties such as Royal Gala, Braeburn, Granny Smith	0.735			
Importance of—Labelled as club apples such as Pink lady or Cosmic Crisp	0.747			
Importance of Apple Physical Attributes		0.723	0.825	0.543
Importance of—Colour of the skin is true to variety	0.773			
Importance of—Smell is appealing	0.700			
Importance of—Texture is soft	0.793			
Importance of—Skin is free of visual blemishes	0.673			
My Attitudes towards US Apple Growers		0.836	0.880	0.552
I think that US growers have a longstanding tradition and lots of experience in growing sustainable apples.	0.728			
I think that US apple growers contribute to the care and maintenance of the landscape	0.678			
I think that US apple growers make active contributions to preserve biodiversity	0.841			
I think that US apple growers treat land resources responsible	0.707			
I think that social pressure on apple growers should be increased as they are main agents of climate change.	0.665			
I think that US apple growers are environmental conscious	0.821			
Subjective Apple Knowledge		0.860	0.905	0.704
I understand a lot about apples	0.821			
I am confident in my knowledge of apples	0.810			
Among my friends I am the apple expert	0.882			
I know more about apples than others do	0.841			

**Table 3:** Scale discriminant validity.

Fornell–Larcker Criterion	Discerning Apple Buyer	Importance of Apple Commercial Attributes	Importance of Apple Physical Attributes	Attitudes towards US Apple Growers	Subjective Apple Knowledge
Discerning Apple Buyer	0.710				
Importance of Apple Commercial Attributes	0.638	0.726			
Importance of Apple Physical Attributes	0.571	0.719	0.737		
Attitudes towards US Apple Growers	0.503	0.476	0.501	0.743	
Subjective Apple Knowledge	0.484	0.426	0.360	0.548	0.839
**Heterotrait–Monotrait Ratio**					
Discerning Apple Buyer					
Importance of Apple Commercial Attributes	0.831				
Importance of Apple Physical Attributes	0.713	1			
Attitudes towards US Apple Growers	0.588	0.614	0.618		
Subjective Apple Knowledge	0.566	0.546	0.417	0.635	

**Table 4:** Path coefficients and hypothesis testing results.

Hypothesised Relationship	Coefficient	T Stat	*p* Value
H1a: Objective Apple Knowledge -> Discerning Apple Buyer	−0.008	0.191	0.848
H1b: Subjective Apple Knowledge -> Discerning Apple Buyer	**0.456**	11.929	0.000
H2a: Gender -> Discerning Apple Buyer	−0.027	0.627	0.530
H2b: Age -> Discerning Apple Buyer	−0.077	1.773	0.076
H2c: Education -> Discerning Apple Buyer	0.068	1.511	0.131
H2d: Income -> Discerning Apple Buyer	−0.054	1.206	0.228
H3a: Objective Apple Knowledge -> My Attitudes towards US Apple Growers	**−0.086**	2.133	0.033
H3b: Subjective Apple Knowledge -> My Attitudes towards US Apple Growers	**0.536**	10.553	0.000
H4a: Gender -> My Attitudes towards US Apple Growers	−0.006	0.129	0.898
H4b: Age -> My Attitudes towards US Apple Growers	0.031	0.729	0.466
H4c: Education -> My Attitudes towards US Apple Growers	**0.126**	2.134	0.033
H4d: Income -> My Attitudes towards US Apple Growers	0.005	0.140	0.889
H5: Discerning Apple Buyer -> Importance of Apple Physical Attributes	**0.428**	7.142	0.000
H6: My Attitudes towards US Apple Growers -> Importance of Apple Physical Attributes	**0.286**	4.776	0.000
H7: Discerning Apple Buyer -> Importance of Apple Commercial Attributes	**0.534**	9.267	0.000
H8: My Attitudes towards US Apple Growers -> Importance of Apple Commercial Attributes	**0.208**	3.586	0.000

Bold = *p* < 0.05.

## Data Availability

The data presented in this study are available on request from the corresponding author.
